# A single-center comparison of our initial experiences in treating penile and urethral cancer with video-endoscopic inguinal lymphadenectomy (VEIL) and later experiences in melanoma cases

**DOI:** 10.3389/fsurg.2022.870857

**Published:** 2022-09-26

**Authors:** A. Gómez-Ferrer, A. Collado, M. Ramírez, J. Domínguez, J. Casanova, C. Mir, A. Wong, J. L. Marenco, E. Nagore, V. Soriano, J. Rubio-Briones

**Affiliations:** ^1^ Urology Department, Valencian Institute of Oncology Foundation, Valencia, Spain; ^2^ Dermatology Department, Valencian Institute of Oncology Foundation, Valencia, Spain; ^3^ Medical Oncology Department, Valencian Institute of Oncology Foundation, Valencia, Spain

**Keywords:** lymph node excision, endoscopy, urethral neoplasms, penile neoplasms, melanoma

## Abstract

**Background:**

Video-endoscopic inguinal lymphadenectomy (VEIL) is a minimally invasive approach that is increasingly indicated in oncological settings, with mounting evidence for its long-term oncological safety.

**Objectives:**

To present our single-center experience of treating penile and urethral cancer with VEIL, as well as its more recent application in melanoma patients.

**Methods:**

We prospectively recorded our experiences with VEIL from September 2010 to July 2018, registering the patient primary indication, surgical details, complications, and follow-up.

**Results:**

Twenty-nine patients were operated in one (24) or both (5) groins; 18 had penile cancer, 1 had urethral cancer, and 10 had melanoma. A mean 8.62 ± 4.45 lymph nodes were removed using VEIL and of these, an average of 1.00 ± 2.87 were metastatic; 16 patients developed lymphocele and 10 presented some degree of lymphedema; there were no skin or other major complications. The median follow-up was 19.35 months; there were 3 penile cancer patient recurrences in the VEIL-operated side. None of the melanoma patients presented a lymphatic inguinal recurrence.

**Conclusions:**

VEIL is a minimally invasive technique which appears to be oncologically safe showing fewer complications than open surgery. However, complications such as lymphorrhea, lymphocele, or lymphedema were not diminished by using VEIL.

## Synopsis

VEIL is a minimally invasive alternative to conventional inguinal lymphadenectomy which appears to be safe for oncological patients with early-stage disease. Compared to open surgery, VEIL produces fewer complications, especially related to the skin. The recurrence rate for penile cancer over 22.75 ± 18.7 months was 8.8%. No melanoma patients treated with VEIL presented in-field lymphatic inguinal recurrences. Complications related to lymphatic drainage remain a problem requiring future investigation.

## Introduction

Penile cancer is a rare malignancy which most commonly affects men aged over 50 years and whose worldwide age-standardized incidence is estimated at 0.84 cases per 100,000 person-years (1). Lymph-node status strongly indicates penile cancer prognosis and survival, thus, early and complete lymphadenectomy to remove lymph-node metastases is crucial to obtain higher curative and survival rates compared to “wait-and-see” policies (2–4). According to European Association of Urology guidelines, histological nodal staging is mandatory for all high grades and/or stages above pT1a (5). This is usually achieved by conventional staging inguinal lymphadenectomy (3) or by dynamic sentinel node biopsy followed by therapeutic lymphadenectomy in cases when a metastatic sentinel node is found (5). However, these procedures have high morbidity rates (20%–80% of cases), with minor complications including limited wound necrosis and infection and major complications including widespread skin dehiscence, cellulitis, skin flap necrosis, lymphoceles, deep vein thrombosis (DVT), thromboembolic phenomena, prolonged hospital stays, nosocomial infections, or even death (6–10). Permanent sequelae such as unaesthetic scarring and chronic leg or penoscrotal lymphedema are also frequent and can sometimes be difficult to manage (11, 12).

Thus, less aggressive diagnostic procedures such as sentinel node biopsy (13) or modified limited lymphadenectomy are becoming more common substitutes. Moreover, because of a lack of experience and fear of the risk of complications, penile cancer patients are sometimes not histologically staged at all and are wrongly managed with surveillance only. Given the aforementioned problems, a new minimally invasive endoscopic technique commonly referred to as video-endoscopic inguinal lymphadenectomy (VEIL) has been developed and ushered in as a welcome addition to surgeons' arsenal of tools for performing inguinal lymphadenectomy. Initial data suggest that, although this endoscopic surgical technique is not completely complication-free (14), it produces less morbidity than open-surgery approaches (15, 16), better cosmetic results (15, 17, 18), and is safe for use in different malignancies involving inguinal lymph-nodes (19, 20). Given that penile cancer is rare, and VEIL has not yet demonstrated reproducible and safe long-term oncology results, VEIL has not yet replaced conventional radical inguinal lymphadenectomy as the standard-of-care treatment. Even though VEIL is becoming more common, in 2012 it was used in no more than 20 centers worldwide (16). Moreover, only 10 published series employing VEIL have been published to date, and these just included 7–32 patients each (21). VEIL procedures are currently routinely performed in only 15 referral hospitals worldwide, as summarized in Table 1 (7, 8, 10, 13, 16, 22–33).

**Table 1:** Video-endoscopic inguinal lymphadenectomy procedures currently routinely carried out in referral centers worldwide.

Surgical approach	City (country)	Author	Year	Primary tumor	Patients	Procedures	Operating time (mins)	No. LNs retrieved	No. Pathological LNs	Drain (days)	Lymphocele or Seroma	Other complications	In-field recurrences	Follow-up in months
VEIL	Sao Paulo (Brazil)	Tobías-Macado	2008	Penile	15	20	120 (90–160)	10.8 (7–16)	4 groins	4.9 (3–12)	2 (10%)	1 (5%)	none	30.5
VEIL	Caracas (Venezuela)	Sotelo	2009	Penile	8	14	60 (50–150)	9 (4–15)	3 patients (4 LNs)	not reported	21.40%	none	1/8 at 12 months	not reported
VEIL	Atlanta (U.S.A.)	Delman	2011	Penile (12); anus (2); melanoma (18)	32	45	165 (75–245)	11 (4–24)	Na	15 (7–25)	2	Cellulitis (2); lymphedema (2)	none	not reported
VEIL-H (abdominal approach)	Chongqing (China)	Xu	2011	Vulvar	17	17	94 (70–150)	16 (11–23)	5 patients	5–8	6	none	0	13
VEIL 36, raVEIL 3	Delhi (India)	Sudhir	2012	Penile (19); vulvar (2); urethra (1)	22	39	na	na	9 patients	not reported	4 (10.2%)	Skin flap necrosis (1)	none	33.4 (8–55)
VEIL	Lucknow (India)	Pahwa	2013	Penile	10	20	120–180	7–12	1 patient (2 LNs)	5.1 (4–8)	2 patients	none	none	3–14
VEIL	Tübingen (Germany)	Schwentner	2013	Penile (14), melanoma, urethra, testis	16	28	136.3 (87–186)	7.1 ± 2.9	1.6 ± 1.9	not reported	7.1		6.60%	55
VEIL	Guangdong (China)	Zhou	2013	Penile and scrotal Paget's disease	7	11	126 (90–180)	12.3 (7–15)	4 patients (6 LNs)	not reported	18.50%	0	0	16.3 (4–27)
VEIL	Ube (Japan)	Ichimiya	2013	Skin neoplasms (2 melanoma, 1 extramammary Paget's disease, 2 squamous cell)	5	5	163 (148–175)	11.2 (8–17)	Na	not reported	None	Wound necrosis (1); lymphedema (2)	none	not reported
VEIL	Atlanta (U.S.A.)	Martin and Delman	2013	Melanoma	40	36 (4 conversions)	181.3 (85–343)	12.6 (3–24)	1.78 (1–9)	19.8	22.50%	Infection (40%); wound necrosis (2.5%)	1 (9.1%)	19.1 months
VEIL	Minneapolis / Rochester (U.S.A.)	Abbott	2013	Melanoma	13	13	245 (205–366)	11 (9–15)	Na	28 days (IQR: 18–45)	38.40%	Wound/skin (7.7%)	0	5
VEIL+ iliac lap	Padua (Italy)	Sommariva	2016	Melanoma	23	20 (4 conversions)	132.5 (106–154)	9.5 (8–14.5)	1 patient	10 (7–21)	7	Infection (4)	2	18
VEIL	Knoxville (U.S.A.)	Landry	2017	Melanoma	10	10	226.5 (123–366)	11.5 (6–17)	Na	not reported	1	Wound infection (2); lymphedema (6)	not reported	not reported
raVEIL 27, VEIL 7	Ann Arbor (U.S.A.)	Rusell	2017	Penile	18	34	141.50 (120–150)	10 (VEIL); 8 (raVEIL)	6 patients	36	2 (11%)	Skin (3); DVT (1)	2	5.5
VEIL	Atlanta (U.S.A.)	Postlewait and Delman	2017	Melanoma (63); genitourinary (39)	102	137	193 (± 50)	11.2 ± 4.6	1.9 ± 1.4	29.1 ± 18.0	42 (30.7%)	Wound infection (65; 47.4%); skin necrosis (13; 9.5%)	8 (17%)	38
VEIL	Jaipur (India)	Yadav	2018	Penile	29	29	163.83	7.6	na	na	3 (10.34%)	Skin necrosis (2); lymphedema (3)	not reported	not reported
VEIL-H, VEIL	Changhsa (China)	Yuan	2018	Penile	72	Leg (70); hypogastric (74)	107 (85–185)	20.6 (12–29)	21 patients (0–6)	4.15 days (3–12)	5 (6.9%)	11 (15%)	5 (regional or distant)	16.2

VEIL, video-endoscopic inguinal lymphadenectomy; VEIL-H, VEIL with a hypogastric approach; raVEIL, robotic assisted VEIL; LN, lymph node.

After the surgical and oncological safety of VEIL was observed in initial studies in penile cancer staging lymphadenectomies (15, 17, 18, 23, 28, 34), some of these pioneering authors expanded its use to penile cancer in cases with cN1 mobile lymph nodes (20) or other diseases requiring inguinal lymphadenectomy such as primary vulvar and urethral cancer (11, 14, 30, 35–42) and melanoma (6, 10, 25, 43). Some centers have recently shown the technical feasibility of robotically-assisted VEIL, although these results seemed to show higher complication rates than for the standard VEIL approach (14, 43–46) and do not appear to be advantageous compared to standard VEIL. A hypogastric VEIL approach with similar results to standard VEIL performed *via* leg incisions has also been described for vulvar and penile cancer (30, 32, 41, 47). One group advocates placing 3–4 trocars in the lower abdominal wall for bilateral VEIL which would also allow pelvic lymphadenectomy to continue, when indicated, without reconfiguring the patient or trocar positioning (33).

Here we present our experience of performing VEIL procedures at our institution, starting with penile cancer staging indications and progressively expanding its use to cases of cN1 penile cancer and pN1 melanoma (after sentinel or percutaneous biopsy). Importantly, the guidelines for melanoma very recently changed so that complete regional removal of nodes is no longer recommended after sentinel node biopsy (48). Only clinically diagnosed metastatic lymph nodes or recurrences after sentinel node procedures are now indications for complete regional lymph-node removal, and thus, represent cases in which VEIL might play a role (48). We report the technical variations we implemented while applying VEIL in order to try to minimize the patient morbidity currently associated with the use of this technique.

## Materials and methods

We prospectively recorded the surgical and oncological data from all our patients operated by VEIL between September 2010 and July 2018. We obtained signed written informed consent from all the participants included in this study. The study was approved by our institutional review board and conforms to the ethical principles for medical research set out in the Declaration of Helsinki. The indication for the use of VEIL was established by the multidisciplinary urology and melanoma committees at our institution. In order to avoid inter-operator variation, all the procedures were performed by one surgeon (A.G.F.) who had 12 years' experience in open inguinal lymphadenectomy and penile cancer surgery at the start of the study.

### Surgical technique

**Figure 1:**
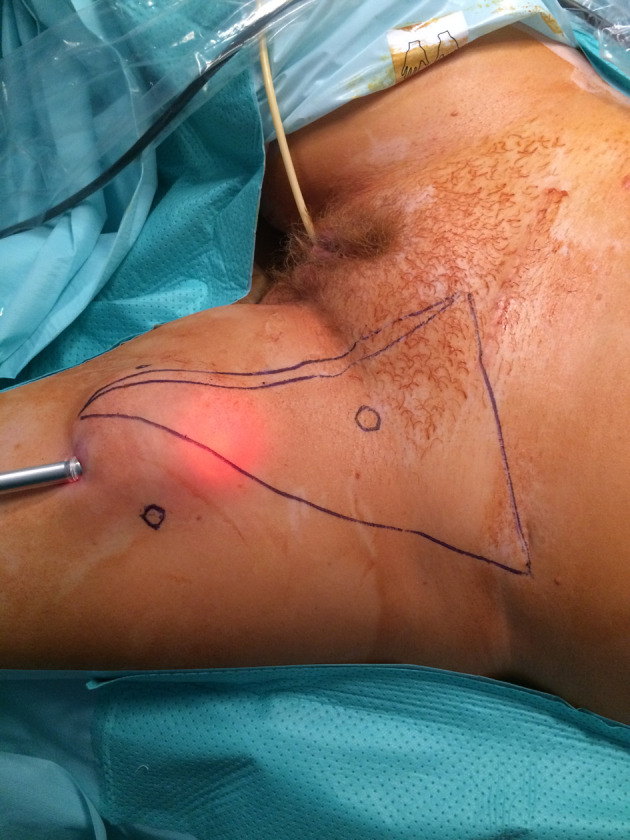
Identification of Scarpa's triangle and trocar positioning sites. The apex of Scarpa`s triangle is the point where the adducto longus (medially) and and sartorius muscle (laterally) meet; its base follows the inguinal ligament line from the pubic tubercle to the anterior iliac spine (distally). The positioning of the trocar sites is shown as circles.

The patients were placed in the supine position with their legs spread to allow the surgeon access to the groin; the laparoscopy screen and insufflator were placed at the patient's shoulder. A small skin incision was made at the inferior tip of Scarpa's triangle up to the level of Scarpa's fascia and, with the aid of a trocar balloon if necessary, a working space was created which allowed the insertion of the camera trocar into this incision (Figure 1). Two trocars were then positioned (11 mm laterally and 5 mm medially), wherever possible in order to reduce lymphedema; we clipped it with Hem-o-loks where this was not possible.

**Figure 2:**
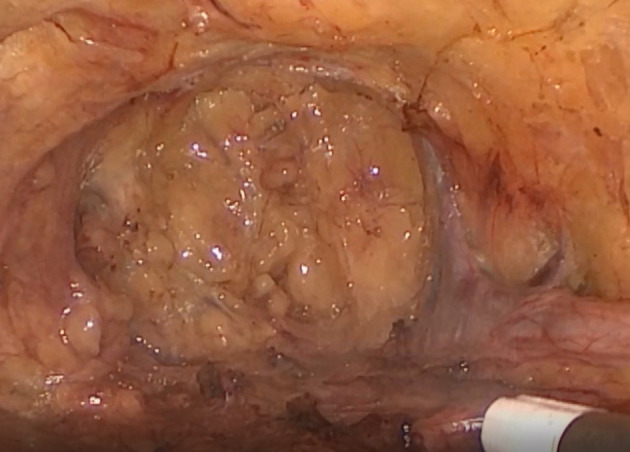
Surgical field after the video-endoscopic inguinal lymphadenectomy procedure. Tisseel fibrin sealants were sprayed onto the surgical field after VEIL was completed.

A meticulous en-block lymphoadipose specimen dissection was performed with the help of laparoscopic vessel-sealing devices, working from the bottom of Scarpa's triangle to the top, sparing the saphenous vein. VEIL is not contraindicated after prior sentinel node biopsy but does make dissection underneath the scar more difficult and so, required care to avoid skin perforations and potential skin necrosis. The lymph node specimens were subsequently removed through the camera port in an endobag. In most cases we ended the procedure by spraying fibrin sealants (Tisseel, Baxter) and placing a suction drain in the surgical field (Figure 2); in two cases we used fibrinogen and thrombin coated sponges (TachoSil®, Takeda) and did not place drainage. After surgery, the leg was wrapped tightly for a week and the patient used compression stockings for 2 months to help prevent lymphedema. Ambulation was started the first day after surgery and the drains were removed when the drain output was lower than 50 cc. We also prevented DVT with low-molecular weight heparin for 3 weeks and gave antibiotic prophylaxis for 5 days.

## Results

We operated a total of 29 patients (34 groins) from September 2010 to September 2017 at our institution: 18 penile cancer cases (1 case also presented prostate cancer which originated incidental inguinal metastases), 1 case of urethral carcinoma, and 10 melanoma patients. We started operating melanoma cases using VEIL in May 2016. The overall mean patient age was 61.5 years (range = 24–81), 4 cases were female, and 25 patients were male. The characteristics of the patients and their VEIL outcomes are shown individually in Table 2 for penile and urethral cancer cases, and in Table 3 for melanoma cases.

**Table 2:** The characteristics of the patients with penile or urethral cancer included in this study cohort.

VEIL	Age	pT stage	cN stage or pN/№ sentinel nodes retrieved	pN (positive/total retrieved)	cN stage or pN+/№ sentinel nodes retrieved	pN (positive/total retrieved)	Lymphedema VEIL	Lymphocele VEIL	Open skin complications	VEIL lymphocele intervention	VEIL recurrence	Open recurrence	Follow-up in months (death)
Unilateral			VEIL side	VEIL side	Open side	Open side							
	54	pT2	cN0	pN0/7	cN3	pN7/12	Yes						23 death
	75	pT1b	pN0/1	pN1/3	pN1/1	pN0/8							92
	43	pT3	cN0	pN0/7	cN2	pN1/10		Yes	Yes	Surgical revision			65
	65	pT2	cN0	pN2/7	cNx	pNx -not performed		Yes		Percutaneous drainage			94
	81	pT3	cN0	pN0/4	cN1	pN0/7	Yes	Yes		Percutaneous drainage			17
	59	pT1b	cN0	pN0/4	cN3	pN8/10		Yes	Yes	Surgical revision	6		16 death
	73	pT1b	cN0	pN3/10	cN3	pT3/6	Yes	Yes	Yes	Surgical revision		13	14 death
	60	pT1b	cN0	pN1/6	cN1	pN8/17			Yes			2	5 death
	78	pT1b	cN0	pN0/7	cN1	pN3/5	Yes		Yes			4	9 death
	51	pT2	cN0	pN0/7	cN3	pN1/1 (conglomerate)		Yes	Yes			3	11 death
	67	pT2	cN1	pN0/5	cN3	pN6/8						5	11 death
+ Prostate cancer	52	pT2	cN0	pN0/6	cN2	pN2/9 prostate mets.			Yes				30
	70	pT2		pN0/6	cN3	pN1/2	Yes					6	15 death
	31	pT2	pN0/1	pN0/7	pN1/1	pN0/9		Yes		Percutaneous drainage	16		33
**Bilateral**			**VEIL side**	**VEIL side**	**Open side**	**Open side**	** **	** **	** **	** **			
	44	pT3	cN0	pN0/12	cN0	pN0/4							48
	62	pT2	cN0	pN1/11	cN0	pN0/13	Left	Yes	Yes	Percutaneous drainage			21
	51	pT2	pN0/1	pN0/9	pN1/5	pN0/5	Left	Yes		Percutaneous Drainage			44
	61	pT2	cN0	pN0/6	cN0	pN0/8	Bilateral	Yes	Yes	Percutaneous Drainage	7		8 death
Urethral cancer	81	pT1	cN0	pN0/9	cN1	pN2/12	Penoscrotal		Yes	Surgical revision			19 death

**Table 3:** The characteristics of the patients with melanoma included in this study cohort.

Age	pT stage	pN+/№ sentinel nodes retrieved	cN+ percutaneous biopsy	pN+/№ nodes retrieved by VEIL	Lymphocele	Lymphedema	Interventions for complications	VEIL recurrence	Follow-up (months)
69	pT4b	PN1/1		PN0/9	Lymphorrhea yes		Percutaneous sclerosis of persistent lymphorrhea		19
72	pT4b		cN1	PN16/27		yes	No action taken		23
78	pT2a		cN1	PN2/7	Yes		Percutaneous drainage		21 (death)
38	pT1a		cN1	PN2/16					32
44	pT1b	pN1/1		pN0/12					28
24	pT2a	pN1/1		pN5/10	Yes		Percutaneous drainage		27
76	pT4b	pN1/1		pN0/11	Yes		Surgical revision		9
72	pT4b	pN1/1		pN0/10					5 (death)
71	pT2a	pN1/1		pN0/11					20
81	pT2a		cN1	pN1/6	Yes		Percutaneous drainage		17

### Video endoscopic inguinal lymphadenectomy patients, procedures, and complications

Comparative data related to the VEIL interventions carried out are shown in Table 4. A total of 5 patients underwent bilateral VEIL: 3 cases of intermediate and high-risk (cN0) penile cancer, 1 case of cN1 urethral cancer (a single mobile lymphadenopathy), and 1 case of pN1 (sentinel-node biopsy) penile cancer. Unilateral VEIL was performed in 24 patients: 14 cases of penile cancer and 10 with melanoma. Of the former, we performed open contralateral-side surgery in 11 cases because > cN1 and because of a positive sentinel node biopsy outcome in 2 cases; 1 patient underwent unilateral side surgery because he had already undergone open-surgery in his other groin at another center. The melanoma cases had histologically proven inguinal metastases diagnosed by percutaneous biopsy in 4 cases and by sentinel-node procedure in the other 6.

**Table 4:** Comparative data of VEIL procedures in penile / urethra vs melanoma cases.

Patients	Procedures	Operating Time (mins) Mean (SD)	Saphenous Vein preservation	Number of lymph nodes removed Mean (SD)	Pathologic lymph nodes Mean (SD)	Complications Lymphocele	Complications Lymphedema	Drainage (days) Mean (SD)	Hospital stay, only VEIL (days) Median (IR)	Recurrences In-field	Median Follow-up Months (IR)
Total 29	34	117.06 (22.90)	16 (47.1%)	8.62 (4.45)	1.00 (2.87)	16 (47.1%)	10 (29.4%)	8.69 (3.86)	6 (5–8.25%)	3 (8,8%)	17.92 (10.2–27)
Penile / Urethral 19	24	121.25 (20.71)	12 (50%)	7.25 (2.80)	0.33 (0.76)	11 (45.8%)	3 (30%)	7.82 (3.20)	9 ( 8.25–13.5)	3 (12,5%)	15.7 (10.5–26)
Melanoma 10	10	107.00 (25.84)	4 (40%)	11.90 (5.97)	2.60 (4.97)	5 (50%)	7 (29.2%)	10.60 (4.62)	5.5 (5–6)	0	19.38 (10.3–25.6)

All the VEIL procedures were completed successfully without intraoperative complications or open-surgery conversions. The median operating time was 120 min (range = 100–127.5 min) and a median of 8.62 (*SD* = 4.45) lymph nodes were retrieved. Ten groins out of the 34 VEIL procedures carried out harbored metastases and a mean of 1.00 (*SD* = 2.87) metastatic nodes were recovered. The mean follow-up time was 22.75 (*SD* = 18.7) for recurrences in VEIL-operated groins and 12 patients died after systemic progression. The saphenous vein was preserved in 18 procedures (52.9%) and the median drain duration was 8 days (range = 6–12).

A total of 15 groins developed lymphocele (4 of them infected) and were managed with percutaneous drainage in 10 cases; 5 patients underwent surgical revision, which was performed simultaneously in 4 patients requiring pelvic lymphadenectomy in order to complete node staging for inguinal node-positive disease. The 10 cases of penoscrotal or leg lymphedema required conservative management only and there were no skin-related complications. A drain was not placed at the end of surgery in 2 cases, although the compression stockings were maintained; one of these cases developed lymphocele and the other did not.

### Penile cancer patients

A median of 7 lymph nodes (range = 5.75–9.0) were removed from the 19 patients diagnosed with ≥pT1b, cN0–cN1–pN1 penile cancer (positive sentinel node in 3 cases) undergoing unilateral or bilateral VEIL procedures. A total of 8 metastatic lymph nodes were identified in VEIL-operated groins in 5 patients, of which contralateral groin metastases were also identified in 2 cases using an open-surgery approach. The mean follow-up of penile cancer patients who underwent a VEIL procedure was 24.2 (*SD* = 22.4) months (range = 5–93). None of the patients with metastatic lymph nodes retrieved by VEIL relapsed in the same groins, but 3 (12.5%) patients with negative nodes had recurrences in the VEIL-operated groin and subsequently died because of progression.

However, we must consider the following peculiarities of these cases: in one, subcutaneous cancerous inguinal spread was diagnosed one year after the VEIL procedure with synchronic local recurrence, although no cancerous lymph nodes were detected in salvage surgery. The other had had 8 positive nodes in the open side with early systemic progression and only 1 patient with only a VEIL side recurrence progressed. Another 8 patients had metastases only in the contralateral side operated by open-surgery; despite undergoing further treatments, 6 of these patients, as well as 1 VEIL-positive and 1 urethral cancer patient, experienced systemic progression and died.

### Melanoma patients

In addition, from May 2016 to November 2017, 10 patients with primary melanoma (7 lower extremity; 1 subungual toe; 1 left abdominal flank; and 1 scrotal) were included in this study. These patients had been diagnosed with metastatic lymph nodes by inguinal sentinel lymph node biopsy in 6 cases and by ultrasound-guided biopsy in the other 4 cases. Moreover, non-systemic metastatic disease was studied with CT and PET scans in all these cases. All these patients were successfully operated by VEIL which resulted in the removal of a median of 11.90 (*SD* = 5.97) lymph nodes. We found 1–16 lymph node metastases in the VEIL specimens obtained from 5 patients and these individuals underwent further adjuvant treatment. Two of these 5 cases received radiotherapy in the operated field and this adjuvant treatment did not result in the development of skin complications; 2 patients subsequently died because of systemic progression. After a mean of 20.0 months (range = 4–32), no recurrences had occurred in the VEIL-operated fields.

## Discussion

In this study we performed a total of 34 penile and melanoma VEIL procedures which resulted in 3 inguinal recurrences (a figure similar to other reports in the scientific literature (11, 14, 35, 47). Most of the patients that progressed and died either had metastases in their open operated fields or developed distant metastases early on. Only one of our pN0 VEIL field patients (who developed a regional recurrence) died from progression; the other patient, who had negative VEIL and negative open lymphadenectomy results, developed subcutaneous inguinal spread synchronically with a local recurrence, and no residual lymph nodes were found in salvage surgery performed in that groin. The third patient with recurrence in VEIL and a high metastatic load in his open operated groin had systemic progression was much earlier on and died for this reason rather than his VEIL recurrence.

It is important to consider that VEIL was initially indicated as a cN0 staging procedure in cases with an inherently lower risk of harboring metastases. Therefore, given that we retrieved an increasing number of cancerous lymph nodes during our learning curve, it is advisable to also closely monitor even low-risk patients. Nonetheless, our mean follow-up time of 24.2 months (range = 5–92 months) appears to be sufficient time to adequately assess the oncological results of VEIL in this setting, given the rapid onset of metastatic disease in penile cancer when metastatic lymph nodes are overlooked. Previous studies report the collection of a wide range of lymph nodes and, in most cases, very few recurrences (0%–17%), and conclude that VEIL is a safe procedure, albeit after follow-up periods that were usually very short (5, 7–9, 21, 24–27).

To the best of our knowledge, VEIL has only been used for melanoma at 7 centers worldwide (6, 8, 9, 12, 22, 49). Some authors have shown that VEIL is a feasible and safe procedure for melanoma patients which presents fewer complications than conventional open-surgery procedures (8, 25). However, because short or no follow-ups were carried out, or cases using VEIL were not recorded separately by malignancy type, the recurrence rate in melanoma was often not reported (Table 1). The largest series and longest follow-up time for VEIL used in melanoma cases performed to date indicated an in-field recurrence rate of 17% (8/48 patients) at a median follow-up of 38 months (9).

In this present study, we did not see any recurrences in the inguinal fields after a median follow-up of 19.38 months, and only two patients died because of systemic progression. In contrast, we observed 3 in-field recurrences among the penile cancer patients. This is perhaps because penile cancer is a more regionally aggressive disease and because we retrieved fewer lymph nodes from penile cancer patients at the beginning of our learning curve with VEIL. In this series, although VEIL produced lower morbidity, complications related to lymphatic drainage including prolonged lymphorrhea, lymphocele, and lymphedema were very frequent. Nonetheless, we did not observe any skin-related complications even though they are relatively common after traditional inguinal lymphadenectomy. Prolonged lymphorrhea is a frequent concern and source of discomfort to many patients. However, prolonged drainage usually leads to infection and early removal seems to facilitate the formation of lymphocele. The time drains are left in place is not commonly reported in the literature, but often seems to range from 4 to 36 days; in this study the drains were present for a mean of 8.69 ± 3.86 days.

Our patients very frequently developed lymphocele (47%) and the rate at which this complication appeared was not related to our expertise in VEIL. The presence of seroma/lymphocele are reported in different ways in the literature although these were common complications (in 0%–38.4% cases). Of note, the complication rates reported for robotically-assisted VEIL are even higher than those for the standard VEIL approach and also seem to produce a high prevalence of lymphocele similar to that of our own results (14, 43–46). We found no references in the literature for the use of sealing agents to avoid lymphorrhea in VEIL procedures. Nonetheless, we tested several sealing agents at the end of each surgical procedure to diminish the formation of lymphocele in primary and revision surgeries, but none were useful, perhaps with the exception of TachoSil®. We used this product in two patients and managed their postoperative care with compression stockings but without a drain; one these patients developed lymphocele and the other did not.

**Figure 3:**
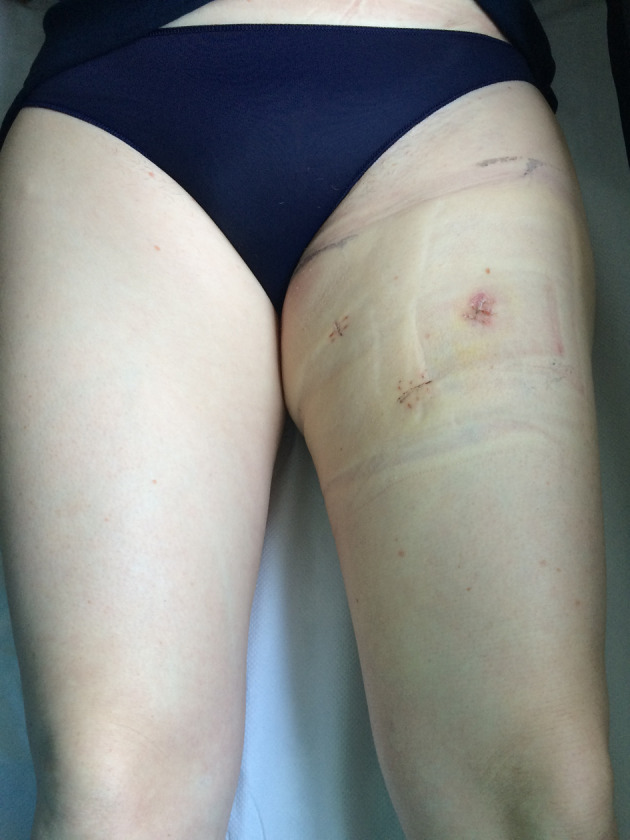
Example of cosmetic result following video-endoscopic inguinal lymphadenectomy in a young patient diagnosed with melanoma.

Similar to open-surgery approaches, lower extremity lymphedema occurred in 10% of cases and penoscrotal lymphedema presented in 1 of the 5 patients who underwent bilateral VEIL but in none of those who received unilateral VEIL. Overall, we did not see any significant differences in the complications presented in patients who had previously undergone sentinel node biopsy, or between melanoma vs. penile cancer sides that were operated by VEIL. Importantly, we observed that complications were generally more frequent and severe in older or obese patients and so it will be important to collect more sociodemographic data in future studies in order to assess these risk-factor correlations. Finally, skin-related complications were rare and the cosmetic results we obtained in our younger patients were excellent, as shown in Figure 3. Therefore, VEIL seems to be a particularly good option for treating patients for whom this issue is a concern.

## Conclusions

VEIL could be a useful alternative surgical approach, especially in penile cancer staging procedures and in melanoma cases when inguinal lymphadenectomy is indicated. Nevertheless, extreme care must be taken when dealing with cN1 and very high-risk penile cancer cases because of the possibility of missing nodes and thus, performing less radical lymphadenectomy. Moreover, we still lack data with longer follow-up periods. We also believe there is some degree of learning curve when gaining experience with VEIL because in this series we obtained an increasing number of lymph nodes as we became more proficient with the procedure. However, we were able to effectively use VEIL to treat melanoma patients with biopsy-proven metastatic lymph nodes without the subsequent appearance of in-field recurrences.

In terms of complications, in our setting, VEIL produced far fewer and less severe skin-related complications than conventional inguinal lymphadenectomy, and like open-surgery procedures, lymph-related events seemed to be the main source of major complications related to VEIL. Thus, in this study lymphorrhea and lymphocele remained an unresolved problem. Nonetheless, in our hands, postoperative recovery after VEIL was shorter and the cosmetic results were much better than those of standard techniques.

There are still a limited number of accepted indications for VEIL and so we recommend these procedures be performed at specialized centers with a high load of potential cases of primary disease with inguinal lymphatic involvement including penile, vulvar, and urethral cancer, melanoma, and other skin malignancies. This would help diminish surgeons' learning curves related to VEIL, therefore encouraging the faster improvement of the surgical and oncological safety of this procedure. Future studies should aim to study sociodemographic risk factors (such as age, smoking habits, and BMI) and procedural risk factors (including saphenous vein preservation, drainage time, operating time, the use of sealing devices, clips, or sealants, and the length of time that compressive dressings or stockings are maintained) that could be associated with VEIL complications. Future work should also assess the effectiveness of this technique in treating patients with different cancer types at a wider range of disease stages, in larger patient cohorts, and in direct comparison with standard contralateral side inguinal lymphadenectomy.

## Data Availability

The raw data supporting the conclusions of this article will be made available by the authors, without undue reservation.
